# Our cities, our farm lands: The socioeconomic determinants of urban households participation in urban agricultural production under climatic stressors

**DOI:** 10.1016/j.heliyon.2024.e35539

**Published:** 2024-08-08

**Authors:** Godwin K. Naazie, Isaac Agyemang, Anthony M. Tampah-Naah

**Affiliations:** a Department of Environment and Resource Studies, Faculty of Integrated Development Studies, Simon Diedong Dombo University of Business and Integrated Development Studies, (SDD-UBIDS) P.O. Box WA64, Wa, West Africa, Ghana; b Department of Mathematics and Natural Sciences, College of Arts and Sciences, William V.S. Tubman University, P.O Box 3570, Harper, Maryland County, Republic of Liberia; c Department of Geography, Faculty of Social Science and Arts, Simon Diedong Dombo University of Business and Integrated Development Studies, (SDD-UBIDS) P.O. Box WA64, Wa, West Africa, Ghana

**Keywords:** Urban agriculture, Climate change adaptations, Agricultural participation, Urban farmers

## Abstract

In Africa, urban agriculture is critical in addressing food security issues, economic and environmental sustainability in rapidly urbanizing regions such as urban Ghana. However, the factors that influence urban residents' participation in urban agricultural production under climate change adaptation has little space in the extant literature. Recognizing the increasing challenges posed by climate change, this study aims to understand the socio-economic factors influencing urban households' participation in agricultural activities and its implications for climate change adaptation and to draw urban households' socio-economic characteristics and their association with participating in urban agricultural production in the era of climate change effects in urban areas of Ghana. A quantitative approach is employed, involving a sample size of 362 urban households' across diverse neighbourhoods. Statistical analyses, including descriptive statistics—frequencies and percentages, inferential statistics—chi-square test and binary regression models, are employed to quantify the relationships between demographic factors and participation levels. The data suggests correlations between demographic variables, such as household size and income are significant at an alpha 0.05 in determining an urban household's participation in urban agricultural production under climatic stressors. Meanwhile, more urban households' in middle and high-class areas participate in urban agriculture than the lower class. Land acquisition is basically through purchasing which is a challenge in urban agriculture production. The study concluded that urban household size and monthly income are influential factors in urban households' participation in urban agricultural production even though land acquisition plays a factor. The study suggests that policymakers and stakeholders should harness the potential of urban agriculture for sustainable development in the era of climate change. This should be done through rolling out pro-poor urban development policies like pro-poor rights and legislation in urban areas; poor access to financial markets; and land tenure reforms that include flexible land holding and access by the poor.

## 1 Introduction

Globally, urban agricultural production has gained significant attention as a means of enhancing food security, and promoting sustainable urban development [1]. In addition, in the presence of global development, where rapid urbanization and environmental challenges intersect, the significance of UA cannot be overstated. As cities expand and the climate becomes increasingly unpredictable, urban households are turning to agricultural production within city limits to buffer against food insecurity and economic instability. Urban agriculture in this study is operations located within cities, and has emerged as a viable means to meet urban food demand through crop cultivation and the raising of animals within urban areas [2]. This is a way to meet sustainable development goals; 1, 2, 12, and 13. For instance, goal 1 – ending poverty across the board, goal 2 – ending hunger, food insecurity, and nutrition and promoting sustainable agriculture, goal 12 – meeting sustainable production and consumption, and goal 13 – minimizing climate impact [3]. Urban agriculture (UA) plays a significant role in climate change adaptation, offering a range of environmental, social, and economic benefits that help urban communities cope with the impacts of climate change [4]. Again, green spaces created by urban farms and gardens help cool urban areas by providing shade and releasing moisture through evapotranspiration at different phases of crop development [5]. Also, plants in urban agricultural settings absorb pollutants like zinc and produce oxygen, contributing to better air quality and mitigating the health of global climatic effects [6]. However, urbanization is one of the challenges to urban agriculture. Urbanization is a global phenomenon, with an increasing number of people residing in urban areas seeking economic opportunities, better living standards, and improved quality of life [7]. However, rapid urban expansion often comes at the cost of environmental sustainability and food security.

In response to these challenges, the concept of urban agriculture has gained prominence, offering a multifaceted solution that goes beyond food production alone—crops and animals [8,9]. Traditionally considered the domain of rural landscapes, agriculture is finding a new frontier within urban spaces, challenging the conventional boundaries of food production [10]. The engagement of urban residents in agriculture not only contributes to local food production enhances environmental sustainability, and promotes a deeper connection between urban dwellers and sustenance in the form of rooftop gardening, community gardens, and aquaponics [11]. Climate change impedes urban agricultural production. Climate change here refers to long-term alterations in global temperature and weather patterns [12]. Aside, from climatic challenges, land accessibility, training, financial support, and developing supportive policies are deterrent for urban households interested in participating in urban agriculture, as in the case of sub-Saharan Africa [7].

Urban agriculture in sub-Saharan Africa, is a potential solution to various challenges faced by urban households, including food insecurity and climate change adaptation [13]. As urbanization continues to increase, the demand for food in urban areas is also on the rise. Urban agriculture presents an opportunity for urban households to actively participate in food production, thereby contributing to food security and resilience against the impacts of climate change. Additionally, existing policies and regulations may not always be conducive to promoting and facilitating urban agriculture within cities, creating barriers for interested households [14]. For instance, urban areas are under city/urban planning restrictions [15]. Urbanization reduces land availability for urban households' participation in urban agriculture production [16]. In addressing the barriers hindering urban households' involvement in agricultural activities and promoting climate-resilient farming practices, cities can harness the potential of urban agriculture as a means to enhance food security and build resilience against climate change [17]. On the policy front, urban households can be empowered to actively engage in urban agriculture while simultaneously adapting to climatic effects on urban crops and animal production [18]. There is a link between urban households' participation in urban agriculture production and urban farmers' climate change adaptation strategies particularly understanding farmers’ sociodemographic factors. As cities continue to expand and populations grow, urban households are increasingly participating in agricultural production within urban areas. This trend not only contributes to local food production but also plays a crucial role in climate change adaptation strategies [19]. As such urban households can adapt to changing environmental conditions and ensure a more secure food supply for themselves and their communities [20,21]. Many cities in the Global South are emerging urban agricultural hubs.

In many rapidly growing cities across the Global South, including those in Ghana, urban agriculture serves as a critical buffer against economic uncertainties and environmental stresses [20]. As climate change continues to exacerbate environmental challenges, urban households are increasingly turning to agricultural production to mitigate the impacts of climatic stressors [21]. However, the decision to engage in urban farming is influenced by a complex interplay of socioeconomic factors. In urban Ghana, climatic stressors come in many forms—erratic rainfall, prolonged droughts as well as increasing temperatures pose significant threats to food security and household welfare [3]. These climatic challenges necessitate adaptive strategies, among which urban agriculture stands out for its potential to provide fresh produce, enhance dietary diversity, and generate supplementary income. Yet, not all urban households participate in this adaptive practice. Understanding the socioeconomic determinants that drive or hinder urban agricultural production is crucial for designing policies that support urban resilience and sustainable food systems.

There are several scholarly works on urban agriculture. For instance, Allen et al. [22] and Mackay [23] looked at the type of crops cultivated in the urban space, Akoto-Danso et al. [24] worked on urban farm nutrient maintenance and Werner et al. [25] emphasized urban farms soil moisture conservation through irrigation. Also, Naazie et al. [26] looked at the spatial distribution of urban agriculture activities, Naazie et al. [27] worked on the characterization of urban agriculture while Naazie et al. [28] explored how urban farmers in secondary cities of Ghana employ climate change adaption strategies in crops and animal production. However, little is done to establish the socio-economic determinants of urban farmers' participation and how that translates to urban farmers’ knowledge acquisition which influence their climate change adaption decision making. Meanwhile, the sociodemographic factors of urban farmers define urban households interest to participate in urban food production adaptation [29]. Urban residents under gardening initiatives can strengthen social ties, exchange knowledge about sustainable farming practices, and collectively address the climatic stressors. This sense of community empowerment is essential for implementing effective climate change adaptations and strategies in urban communities. This study is anchored by these research questions.

1. What are the socioeconomic determinants of urban households' participation in urban crop and animals' production under climatic stressors?
2. How do urban households' socio-economic characteristics associate with their participation in urban crop and animal production in the era of climate change effects?

The study therefore explores the socioeconomic determinants of urban households’ participation in urban agricultural production, aiming to understand the motivations, challenges, and implications of integrating agriculture into the urban fabric. In examining the factors influencing individuals' decisions to participate in urban agriculture, we can uncover valuable insights into the socio-economic variables of urban households and how they determine urban agriculture participation in the era of climate change.

## 2 Conceptualization of urban residents’ participation in urban agricultural production

Urban households’ participation in urban agriculture has interconnectedness between their socioeconomic characteristics. They are categorized below:

***Independent Variables* –** these variables consist of the socio-economic variables of the urban households, encompassing household heads age, gender, income, education, and household size may influence the level of participation in food production within the urban space and this defines farmers' ability to cope with climatic stressors. Again, urban farmers’ socio-economic variables influence both participation in urban agriculture and the implementation of climate change adaptation strategies.

***Dependent Variables* –** among these variables is involvement in urban agricultural production, which gauges how much urban dwellers work in agriculture within the confines of their city. The methods and techniques used by urban residents to participate and to adjust to the effects of climate change, such as variations in temperature, precipitation patterns, and extreme weather events, are reflected in the strategies for adapting to these changes. These constitute another significant dependent variable. The institutional support structure and policies are the mediating variables. The relationship between engaging in urban agriculture production and the successful use of climate change adaptation plans in urban agriculture practice may be moderated by existing supportive laws, rules, and institutional frameworks.

***Hypothesized Relationships in urban agriculture participation and climate change adaptation strategies implication* –** demographic factors are thought to have a favourable impact on urban agriculture participation, while the agricultural response to climate change influences understanding and resource accessibility. Urban agricultural engagement, expertise, and resource accessibility are predicted to have a favourable impact on strategies for adapting to climate change. It is hypothesized that involvement in urban agriculture and the successful application of measures for adapting to climate change are mediated by policy and institutional support. The suggested correlations are tested and validated through empirical study using this conceptual framework as a guide. Policymakers and academics may create focused interventions to support sustainable urban farming practices and improve community resilience in the face of climate change by having a thorough understanding of the factors influencing involvement in urban agriculture and strategies for adapting to climate change.

## 3 Materials and methods

### 3.1 Location, size, and demographics of wa municipal

**Fig. 1:**
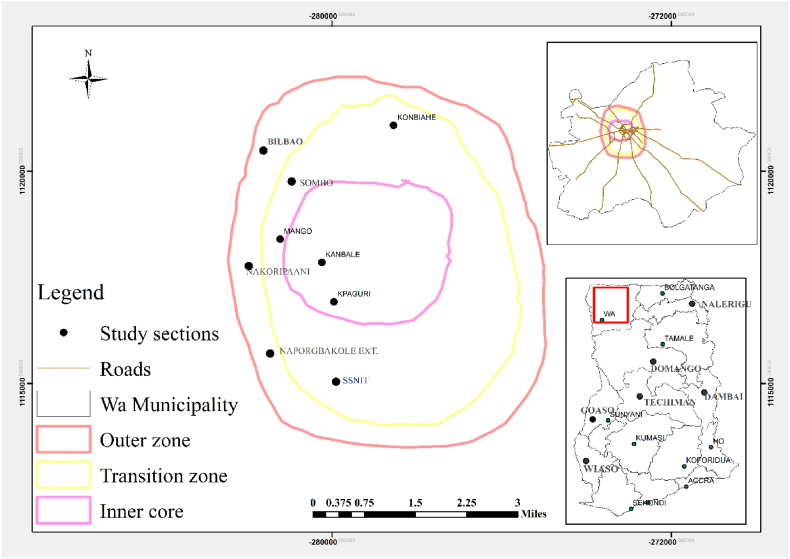
Study area map.

**Source:** Adopted from Naazie et al. [27].

The study took place in the Upper West Region of Ghana using the Wa Township found in the Wa Municipality which lies between 1°40′N to 2°45′N and longitudes 9°32′ to 10°20′W on the globe [30]. According to Ghana Statistical Service [29], the municipality landmass is about 234.74 km square and the population stands at 200,672 people consisting of males—98,493 (49.0 %) and females 102,179 (51 %). The statistics indicated above reflect increased urban demand though urban expansion affects urban food production [31]. This is worsening as climate change and variability stressors affect urban farming activities. The demographics of the population show that about 75.7 % of the people have attained a formal educational level while 6.6 % have primary education, and 17.0 % have secondary and tertiary education [32]. Also, the average urban household size is about 5 persons in a household as well as 49 % of the people with age not less than 18 years up to 60 years, 47 % are less than 18 years and 4 % are aged—beyond 60 years [30]. This means that the level of education of the people living in the Wa township could influence the desire of households to produce either crops or animals or both in the urban area. The study covered almost all the sections in the Wa township. The study classified the Wa township into three (3) zones—low class, middle class, and high classes using emphasis from Osumanu et al. [33]. From Fig. 1, the inner part of the city, thus Kanbale, Kpaguri, and Mango is a low class, the transitional— Sombo, Konbiehi, and Social Security and National Insurance Trust (SSNIT) is a middle class and the outer zone like Naporgbakole, Nakoripaani, and Bilbao is the high class areas.

There are varying levels of employable opportunities in the municipality. Approximately 70 % of the workforce is employed in the agricultural sector, with the remaining 18 % going to various forms of economic activity, industry (3 %), and commerce (9 %). Just 30.2 % of individuals are involved in the production of food crops and animals, despite the fact that 70 % of people work in agriculture. The sources of income for households vary depending on the type of economic activity they engage in Ref. [29]. Farmers in the municipality generally cultivate staple food crops like are cereals, legumes, tubers, and vegetables. Inhabitants in the study area rear animals on a subsistence basis such ass sheep, goats, cattle, and pigs, Others are chicken,turkey, ducks, guinea fowls [29,34].

The Wa Municipality's topography is generally undulating, with an average elevation of 160–300 m above sea level. The Wa Municipality's agricultural operations are influenced by the highlands and lowlands that make up its undulating landscape. Furthermore, the vegetation is primarily grass-covered guinea savanna forest, with acacia, ebony, neem, mango, shea tree, and dawadawa, baobab trees scattered throughout. Even though the vegetation appears green during the rainy season, bush fire typically causes the grasses to dry up and the trees to lose their leaves during the dry season [30]. Consequently, the dry season has an impact on crop cultivation, forcing farmers to concentrate on raising livestock. The municipality has bad weather, with a brief, stormy wet season followed by a windy, extremely hot dry season. The months of November through December and January through February are considered the dry season. Between November and February, the North-East Trade Wind (NETW), which comes from the Sahara Desert, brings chilly, dry air (harmattan) [29]. This comes after a sweltering March–April season. The temperature is in the 400–450C range. Animal rearing is widespread during the months of November through April, however agricultural cultivation is not supported during this time. On the other hand, animal death rises during the harmattan season [30]. The wet season lasts from late April to October, with a protracted drought ending in June after torrential rains and flooding. Crop cultivation and animal rearing are impacted by this.

### 3.2 Research approach and design

The study employed quantitative methods of data collection and analysis. According to Tufford and Newman [33] and Gioia et al. [34]**,** employing the quantitative approach gives statistical analysis and understanding. Evidently, the quantitative approach helped give an understanding of the study from the statistical point [35]. The study was anchored by a cross-sectional survey which is good for taking responses from a sample drawn from a larger population at once [36]. Thus, the cross-sectional survey is important as it gives a snapshot view of the responses as compared to a longitudinal survey. The findings of the sample are therefore generalized to represent the entire population. The study population includes all the households within the targeted urban setting. This implies that all the households within the target urban space were involved in the survey. In all, a preliminary survey (listing exercise) undertaken by the researcher showed that there are about one thousand four hundred and fifty (1,450) households that fall within the demarcated communities for the study in the Wa township. The listing was done in a serpentine order. Serpentine order here means a listing process that follows a sequential order from the lower left to the up left end and the same order is followed till the target area is exhausted. This approach is faster as it enables a complete listing of all the houses and households [30]. In selecting the respondents (households) this study employed probability—simple random sampling [37].

### 3.3 Sample size determination

The portion of respondents that is utilized to represent the full study population is known as the sample size [38,39] Sample size determination is crucial in every study because it makes the research process and findings scientific [40,41]. The population that was gathered during the study's listing stage was used to calculate the study sample size. Within the research jurisdiction, there were 1450 households registered, comprising both those that participated in urban production activities and those that do not. The study used Yamane [42; 886] sample size formula to drive at the sample size. This is given as;

[1] $$n=\frac{N}{1+N\left(e\right)2}$$

where:

n = study sample size.

N = sample population.

1 = constant

*e* = 0.05 margin of error.

As such,

N = 1450

*e* = 95 % or an error margin of 0.05 confidence level.

$$n=\frac{1,450}{1+1,450\left(0.05\right)2}$$

$$n=\frac{1,450}{1+1,450\left(0.00025\right)}$$

$$=\frac{1,450}{4}=362.5=362$$

Consequently, a sample size of 362 households was chosen for the research. This was distributed proportionally across all the low, middle, and high clusters (See Table 1).

**Table 1:** Sample Frame and sample size distribution.

Zone	Section/Community	Households total	Number sampled
Inner	Kanbale	102	25
	Kpaguri	125	31
	Mangu	140	35
	**Total**	**367**	**91**
Transition	Sombo/Chorkor	164	41
	Konbiehi	149	37
	SSNIT	175	44
	**Total**	**488**	**122**
Outer	Naporgbakole Ext.	198	49
	Nakoripaani	196	48
	Bilbao/airstrip	201	52
	**Total**	**595**	**149**
**Overall Total**		**1450**	**362**

### 3.4 Methods and tools of data collection

**Survey *method:*** The research utilized a household survey approach to gather quantitative data. This is cost-effective and does not consume time [37] though, the study could have employed a census approach that involves enumerating the target research population which increases cost and time spent [36]. The method of data collection includes a households' face-to-face survey. The head of the family, spouses, and any older person with extensive knowledge of their urban agriculture and animal production activities were the survey respondents. Computer Assisted Personal Interviewing (CAPI), a systematic questionnaire administration method, was used to accomplish this. The questionnaire is broken down into two primary sections: one for agricultural production in urban areas and the other for urban animal production activities. The questionnaire was built on an Android platform using the Kobo toolbox and uploaded onto tablets for the actual households’ enumeration. Lakshminarasimhappa [39] has outlined the advantages of using Kobo toolbox in Computer Assisted Interviewing (CAPI). Firstly, the use of computer-assisted interviewing is very important as it is faster and more convenient as compared to of manual Paper Assisted Interviewing (PAPI) especially using the Kobo toolbox software. Secondly, the use of CAPI is faster compared to PAPI because interviewing is easy and there is no need to enter data into software for analysis after data is collected. The third point is that the data is refined and generated in a soft copy form as soon as the interviews are done and synced to the server. Lastly, the use of CAPI reduces the cost of printing the questionnaire though tablets were rented. On the field, the various households were therefore identified by their popular names and numbers taken during the listing phase for easy tracing to those households. Face-to-face interviews are the most effective way to gather survey data since they provide the possibility of pushing for clarification [40]. However, since many of the respondents are illiterate and the research assistants must translate the questionnaire from English to “Dagaare,” self-administered interviews may be employed instead.

### 3.5 Methods of data analysis and presentations

To prepare them for additional statistical analysis, the household data collected through the survey were revised and improved. Because it provides a numerical comprehension of the research findings, statistical analysis is crucial [41]. Because of this, the study used the Statistical Package for Social Sciences (SPSS version 20.0) to analyze the quantitative data. In this, chi-square tests, cross-tabulations, frequencies, and percentages were used as descriptive statistics and binary logistics regression models were used for additional analysis. Tables, graphs, and figures were used to display the results.

#### 3.5.1 Estimation of binary logistic models

As part of the quantitative analysis, the study intended to use a binary logistic regression model to estimate some demographic features that influence urban residents' participation in crop and animal production. This model was used to determine the factors that influence the decision to participate in crops and animal production in an urban neighbourhood. This offered an opportunity to examine households’ characteristics that can best explain their participation in crop and animal production in urban neighbourhoods. The respondents (household heads) were asked to respond to questions with two responses, pre-coded 0 and 1 for yes or no. To facilitate the modelling process, the binary model used the values 0 and 1. This allowed for the odds ratio to be obtained using a large number of explanatory variables. Furthermore, the researcher got the opportunity to use continuous variables at the same time. This made the binary logistic regression model user-friendly as compared to that of the linear regression model in the scientific fields [42]. The study adopted Gujarati and Porter [43] regression model that gives the following logistic distribution function;

[2] $$\rho_{i}=\frac{1}{1+e^{-\omega_{i}}}+\frac{e^{\omega_{i}}}{1+e^{\omega_{i}}}$$

where: $\rho_{i}$ represents the probability of a household engaging in crop and animal production in the urban neighbourhood.

[3] $$\omega_{i}=\beta_{0}+\beta_{i}\pi_{i}$$

where: $\pi_{i}$ = Vector of explanatory variables.

$\beta_{0}$ = Vector of a constant term.

$\beta_{i}$ = Vector of the logistic regression coefficient.

Given that, ω sorts from ∞ to ∞, $\rho_{i}$ sorts from 0 to 1 and where the probability of not engaging in urban agriculture is $1-\rho_{i}$.

Where:

[4] $$1-\;\rho_{i}=1-\;\frac{e^{\omega i}}{1+e^{\omega i}}$$

in simplifying equation (1) gives;

[5] $$\ln\left(\frac{\rho_{i}}{1-\;\rho_{i}}\right)=\beta_{0}+\beta_{i}\pi_{i}+\infty$$

Again, the probability that a household would engage in crop and or animals’ production in the urban neighbourhood is related to each of the explanatory variables and is given in terms of marginal effect;

[6] $$\frac{\partial \left(\rho i\right)}{\partial \left(\rho i\right)}=\beta_{i}\left[\bar{\rho }\left(1-\;\bar{\rho }\right)\right]$$

Given the dependent mean variable as $\bar{\rho }$ , households engaging in crop and animal production in urban centers is empirically be given as;

[7] $$\ln\left(\frac{\rho_{i}}{1-\;\rho_{i}}\right)=\beta_{0}+\beta_{1}AGE+\beta_{2}SEX+\beta_{3}MARIT_{STAT}+\beta_{4}EMPLOY_{STAT}+\beta_{5}EMPLOY_{TYPE}+\beta_{6}EDU_{STAT}+\beta_{7}RET_{STAT}+\beta_{7}RET_{STAT}+\beta_{8}HH_{SIZE}+\beta_{9}\;HH_{INCOME}+\varepsilon$$

#### 3.5.2 Specification of the socio-economic and demographic features of the respondents

The specification of the socioeconomic variables meant for running the regression model is found in Table 2 below.

**Table 2:** Variables definition and unit of analysis.

Variable	Definition	Unit of Analysis
**Dependent Variable (Y)**	Participation in UA	0 = Yes
		1 = No
**Independent Variables (X)**		
1. AGE	Age in complete years	0 = 18–30
		1 = 31–40
		2 = 41–50
		3 = 51–60
		4 = 60+
2. SEX	Gender	0 = Male
		1 = Female
3. MARIT_STAT	Marital Status	0 = Never Married
		1 = Married
		2 = Separated
		3 = Widow/Widower
4. EMPLOY_STAT	Employment Status	0 = Employed
		1 = Unemployed
5. EMPLOY_TYPE	Employment Type	0 = Formal
		1 = Informal
6. EDU_STAT	Educational Status	0 = No Formal Education
		1 = Basic Education/Middle School
		2 = SHS/Secondary/Vocational
		3 = Tertiary
7. REST_TYPE	Resident Type	0 = Natives
		1 = Settlers
8. HH_SIZE	Household Size	0 = 1–5
		1 = 6 or more people
9. HH_INCOME	Household Monthly Income (GHS)	0 = < 1, 000.00
		1 = > 1. 000.00

## 4 Results

### 4.1 Socio-economic features of urban household heads

According to study results, approximately 39.0 % of urban food producers are between the ages of 31 and 40. Those who fall into the age group of 41–50 years old, or 38.1 %, watch this closely. Those who fall into the age group of 61 years and above make up the least number of responders (2.8 %). The results further revealed that there are more male urban farmers than females representing, about 71.0 % and 29.0 % respectively. Furthermore, the majority of the respondents are married, representing about 73.8 %, 15.7 % never married, and only 3.6 % of them are separated (See Table 3).

**Table 3:** Socioeconomic features of urban household heads (N = 362).

Socioeconomic Features	Frequency	Percent
Age in complete years		
18–30	42	11.6
31–40	141	39.0
41–50	138	38.1
51–60	31	8.6
61 +	10	2.8
**Gender**		
Male	257	71.0
Female	105	29.0
**Marital Status**		
Never Married	57	15.7
Married	267	73.8
Separated	13	3.6
Widow/Widower	25	6.9
**Employment Status**		
Employed	299	82.6
Unemployed	63	17.4
**Employment Type**		
Formal	153	42.3
Informal	209	57.7
**Educational Status**		
No Formal Education	65	18.0
Basic Education/Middle School	43	11.9
SHS/Secondary/Vocational	108	29.8
Tertiary	146	40.3
**Resident Type**		
Natives	92	25.4
Settlers	270	74.6
**Household Size**		
1–5 Members	231	63.8
More than 5 Members	131	36.2
**Household Monthly Income (GHS)**		
0 = < 1, 000.00	196	54.1
1 = > 1. 000.00	166	45.9

Evidentially, the employment status is such that, the majority of the respondents are employed, representing about 82.6 % and only 17.4 % are unemployed. About 57.7 % of the respondents are engaged in the informal sector and about 42.3 % are working in the formal sector. Interestingly, the respondents have attained either a tertiary education level or second cycle level which is representing about 40.3 % and 29.8 % respectively. Only 18.0 % had no formal education and 11.9 % had some basic education. The results had it that the majority of urban farmers are settlers, representing about 74.6 % while only 25.4 % are natives. The majority of the respondents' households have between 1 and 5 members representing 63.8 % while those with 6 or more members are about 36.2 %. It was also clear that about 54.1 % of the respondents' households’ monthly income is less than GHS1,000.00 and those having monthly income beyond GHS 1000.00 are 45.9 % (See Table 3).

### 4.2 Urban dwellers participation in urban food production

To get a deeper understanding of urban residents' participation in agricultural production, the study results showed that, 52.8 % of the respondents were into urban agriculture while 47.2 % were non-participants in urban agricultural food production. Overall, urban food producers are found in the medium and high-class residential zones; about 42.4 % are living in the medium-class zone and the high-class zone is about 41.9 % (See Table 4). Urban agricultural activities take place on purchased lands, family or relatives' land, land acquired as gifts, and owned land, representing about 52.3 %, 32.2 %, and 8.5 % respectively. Interestingly, the results state that about 63.4 % of urban farmers’ activities are affected by land accessibility and only 10.8 % are not affected by accessing agricultural land. Likewise, the main purpose of acquiring lands that are now used for food production in the urban setting is for residential which the majority of urban farmers (82.0 %) attested to. Those who acquired farm plots for agriculture are only about 15.0 % while not more than 1.0 % of lands acquired for either educational or commercial purposes are used for urban cultivation (See Table 4).

**Table 4:** Urban households participation in urban agriculture.

Households participation in urban agriculture	Frequency	Percent
**Yes**	191	52.8
**No**	171	47.2
**Participation in UA by Residential Zone**		
Low Class	30	15.7
Medium Class	81	42.4
High Class	80	41.9
**Total**	191	**100.0**
**Land tenure arrangements in UA**		
Owned Land	2	1.0
Family/Relatives	61	31.2
Purchased	100	52.3
Gift	10	5.2
Rent	16	8.3
Others	2	1.0
**Total**	**191**	**100.0**
**Accessing land for UA**		
Easy to access	21	10.8
Difficult to access	121	63.4
No idea	49	25.8
**Total**	**191**	**100.0**
**The main purpose for acquiring land now used for UA**		
For residential purpose	157	82.0
For farming purpose	30	15.0
For educational purpose	2	1.0
For commercial purpose	2	1.0
**Total**	**191**	**100.0**

The study investigates the relationship between urban households’ socioeconomic characteristics and their involvement in urban food production in order to provide a fuller understanding of the engagement of urban residents in urban farming. The goal is to determine whether urban households' involvement in urban food production is significantly correlated with their socioeconomic characteristics. Cross-tabulation and chi-square independent association testing were used for the analysis (See Table 5). The data indicated that, of the 191 respondents who are involved in urban food production, the majority are between the ages of 31 and 40, accounting for approximately 40.8 % of the sample. This group is closely followed by those between the ages of 41 and 50, which also comprise approximately 37.9 %. Once more, at an alpha level of 0.05, a chi-square test on the relationship between respondents' age and their involvement in urban food production revealed a statistically significant link (p-value = 0.016 < 0.05).

**Table 5:** Association Between Socio-economic Variables and Participation in UA production.

Participation in UA	Age of respondents – Frequency (%)					
	18–30	31–40	41–50	51–60	61+	Total
Yes	17(8.9)	78(40.8)	71(37.2)	15(7.9)	10(5.2)	191(100.0)
No	25(14.6)	63(36.8)	67(39.2)	16(9.4)	0(0.0)	171(100.0)
**Total**	**42(11.6)**	**141(39.1)**	**138(38.1)**	**31(8.5)**	**10(2.7)**	**362(100.0)**
$N=362,x^{2}=12.200,df=4,p=0.016$						

**Participation in UA**	**Gender of respondents – Frequency (%)**		
	*Male*	*Female*	*Total*
Yes	139(72.8)	52(27.2)	191(100.0)
No	118(69.0)	53(31)	171(100.0)
**Total**	**257(80)**	**105(20)**	**362(100.0)**
$N=362,x^{2}=0.622,df=1,p=0.430$			

**Participation in UA**	**Marital status of respondents – Frequency (%)**				**Total**
	*Never Married*	*Married*	*Separated*	*Widow/Widower*	
Yes	24(12.5)	149(78.0)	2(1.0)	16(8.4)	191(100.0)
No	33(19.2)	118(69.0)	11(6.4)	9(5.2)	171(100.0)
**Total**	**57(15.7)**	**267(73.7)**	**13(7.6)**	**25(14.6)**	**362(100.0)**
$N=362,x^{2}=12.143,df=3,p=0.007$					

**Participation in UA**	**Employment status of respondents – Frequency (%)**		
	*Employed*	*Unemployed*	*Total*
Yes	153(80.0)	38(20.0)	191(100.0)
No	146(85.3)	25 (14.7)	171(100.0)
**Total**	**299(83.0)**	**63(17.0)**	**362(100.0)**
$N=362,x^{2}=1.747,df=1,p=0.186$			

**Participation in UA**	**Type of employment – Frequency (%)**		
	*Formal*	*Informal*	*Total*
Yes	91(47.6)	100(52.4)	191(100.0)
No	62(36.2)	109(63.8)	171(100.0)
**Total**	**153(42.2)**	**209(57.6)**	**362(100.0)**
$N=362,x^{2}=12.200,df=4,p=0.016$			

**Participation in UA**	**Educational status of respondents – Frequency (%)**				
	*No Formal Education*	*Basic School*	*Secondary school*	*Tertiary*	*Total*
Yes	37(19.3)	21(10.9)	48(25.1)	85(44.5)	191(100.0)
No	28(16.3)	22(12.8)	60(35.0)	61(36.2)	171(100.0)
**Total**	**65(17.9)**	**43(25.1)**	**108(29.0)**	**146(40.0)**	**362(100.0)**
$N=362,x^{2}=5.460\;df=3,p=0.141$					

**Participation in UA**	**Residential status of respondents – Frequency (%)**		
	*Natives*	*Settlers*	*Total*
Yes	50(26.0)	141(74.0)	191(100.0)
No	42(24.5)	129(75.5)	171(100.0)
**Total**	**92(25.4)**	**270(74.6)**	**362(100.0)**
$N=362,x^{2}=0.124,df=1,p=0.724$			

**Participation in UA**	**Household size of respondents – Frequency (%)**		
	*1 – 5 people*	*More than 5 people*	*Total*
Yes	83(43.4)	108(56.6)	191(100.0)
No	113(66.0)	58(34.0)	171(100.0)
**Total**	**196(54.1)**	**166(45.9)**	**362(100.0)**
$N=362,x^{2}=18.604,df=1,p=0.001$			

**Participation in UA**	**Monthly income status of respondents (GHS) – Frequency (%)**		
	*< 1, 000.00*	*> 1000.00*	*Total*
Yes	115(60.1)	76(30.9)	191(100.0)
No	116(67.8)	55(32.2)	171(100.0)
**Total**	**231(63.8)**	**131(36.2)**	**362(100.0)**
$N=362,x^{2}=2.273,df=1,p=0.132$			

Comparatively, concerning participants’ marital status and their participation in urban food production, about 78.0 % are married and the chi-square test showed a significant relationship between urban household heads marital status and their participation in urban food production. This was shown at an alpha level of 0.05 (p-value = 0.007 < 0.05). On one hand, narrowing down to the type of employment, the urban household heads who engaged in the informal sector are about 52.4 % while those employed by the formal sector are about 47.6 % which is statistically significant given an alpha level of 0.05 (p-value = 0.016 < 0.05). On the other hand, households with a size of more than 5 people indicating about 56.6 % go into urban farming more than households with a membership of less than 5 people which is about 43.4 %. Further analysis indicated that there is a statistically significant relationship between the number of people per household and a member of such household participating in urban food production (p-value = 0.001 < 0.05). About 60.1 % of the urban household income is > GHS 1000.00 while income that is < GHS 1000.00 is about 39.9 % given an alpha level of 0.05, where the p-value = 0.132 > 0.05) (See Table 5).

### 4.3 Socio-economic determinants of participation in urban food production

The study's binary logistic regression model of the urban households' socioeconomic characteristics which affect whether urban households engage in urban agriculture is presented in this section. The Age, marital status, type of occupation, level of education, size of household, and monthly income of the urban household heads are the characteristics of the respondents that were used. To ascertain which of these households' characteristics influences urban households' engagement in urban agriculture production, this was analyzed using the odds ratio and p-values. According to the results, urban household heads who are 60 years of age or older, 31–40 years old, 41–50 years old, and 51–60 years old are, respectively, 0.3, 0.4, 0.5, and 0.6 times more likely to engage in urban agricultural production than household heads who are between the ages of 18 and 30 (See Table 6).

**Table 6:** Socioeconomic determinants of urban households participation in UA production.

Predictors	Urban Households' participation in UA production		
	Odd-ratios	P-value	Standard Error
**Age**			
18–30 (**Reference Category**)	1.00		
31–40	0.431	0.742	0.868
41–50	0.461	0.627	1.251
51–60	0.605	0.461	1.562
61 +	0.267	0.999	0.000
**Marital Status**			
Never Married (**Reference Category**)	1.00		
Married	0.657	2.168	0.239
Separated	0.565	1.324	0.619
Widow/Widower	0.960	11.813	0.010
**Employment Type**			
Formal (**Reference Category**)	1.00		
Informal	1.112	0.756	0.342
**Educational Level/Status**			
No Formal Education (**Reference Category**)	1.00		
Basic Education/Middle School	0.647	0.346	0.462
SHS/Secondary/Vocational	0.930	0.865	0.425
Tertiary	1.430	0.283	0.333
**Household Size**			
1–5 members (**Reference Category**)	1.00		
More than 5 members	0.497*	0.011	277
**Household Monthly Income (GHS)**			
<1, 000.00 (**Reference Category**)	1.00		
>1. 000.00	3.312**	0.001	0.293

**p ≤ 0.01 *p ≤ 0.05.

By statistical standards, the findings are not significant (p-values >0.05 for 0.999, 0.742, 0.627, and 0.461). The aforementioned findings suggest that there is no correlation between urban household heads' ages and their involvement in urban agriculture. Again, the results from Table 6 indicate that respondents who are either separated, married, or widows/widowers are 0.6, 0.7, and 1.0 times more likely to participate in urban agricultural production respectively than those who are single. This indicates an insignificant statistical relationship (p-value = 1.324, 2.168, and 11.813 > 0.05). Similarly, respondents working in the informal sector had a 1.1-fold higher likelihood of engaging in urban agriculture than those in the formal sector. The findings portrayed an insignificant statistical analysis (p-value = 0.756 > 0.05). Urban households' participation in urban agriculture is not determined by their type of employment. In terms of the educational status of the respondents, the analysis shows that household heads who have attained basic/middle school, senior high school (SHS)/secondary/vocational schools, and tertiary are 0.6, 0.9, and 1.0 times more likely to engage in urban agriculture than those who have no formal education. However, the results are insignificant (p-value = 0.3, 0,865, and 0.283 > 0.05) (See Table 6). This means that urban household heads' educational status does not determine one's participation in urban agriculture. In addition, evidence revealed that urban households with more than 5 members are 0.5 times more likely to participate in urban agriculture than urban households with members between 1 and 5. Given the p-value of 0.011 < 0.05, this is significant. The findings indicate that an urban household's engagement in urban agricultural output is influenced by the size of the household. According to additional data, the likelihood of urban families engaging in urban agriculture is 3.3 times higher for those with monthly incomes > GHS1000.00 (GHS 1 = USD 11.43) than for those with monthly incomes < GHS1000.00. This is highly significant (p-value = 0.000 < 0.05) (See Table 6).

## 5 Discussions of the results

The study unravelled the socio-economic factors that determine urban households’ participation in urban agriculture using binary logistics analysis. The findings indicate a significant level of urban residents' engagement in agricultural activities, ranging from small-scale home gardening to community-based initiatives. The data suggests correlations between demographic variables, such as age, income, and education, and the extent of participation in urban agriculture.

### 5.1 Socio-demographics of the respondent

The study findings point to young people who are household heads participating in urban agricultural production than the older household heads. This result is similar to the work of [44] who indicated that older people are less likely to engage in farming, especially in irrigation agriculture. However, the study disagreed with the assertion by Thebe [45] that young people do not participate in agriculture productivity but rather have an interest in formal sector work. The results again do not agree with the work of Albore [42] who asserted that farmers' age determines the increases in production as older farmers are experienced which translates to production. In employment aspects, urban residents who are employed in food production within their homes are affected by the time factor. A study by Bollang and Osumanu [46] revealed that people who are employed especially within the formal sector do not participate in urban food production. Generally, about 16.8 % of the urban population is employed in the formal sector, leaving the remaining 80.2 % in the informal sector [30] and this is not a surprise that formal sector jobs are limited and competitive in most urban settings.

### 5.2 Urban dwellers participation in urban food production

Urban farming is dominating in the high and medium classes within the urban neighbourhood. This is an indication that residents within the medium and high-class residential zones in the cities have more interest in urban food production because of the availability of space than the low-class residential zones even though land availability is general across. This supports that urban agricultural production such as livestock occurs in the inner cities [47]. The implication is that high-class residents are more likely to pay attention to urban food production followed by the medium-class zone than the low-class zone because there is the possibility of almost all being educated and formal workers [48]. Eventually, land accessibility is critical in urban households’ participation in urban agriculture. This is in line with the claim that urban agricultural production is affected by land availability and accessibility as land acquisition is mainly by purchasing [49]. This supports the finding that land ownership, availability, and accessibility influence sustainable agricultural production [50]. As such, extant literature points to a positive relationship between land ownership and successful agricultural production despite climate influence [51].

The study findings showed that the age of the head of the urban household influences their participation in urban agriculture. In line with this, Lawson et al. [52] and Marie et al. [53] said that the age of an individual determines their decision to engage in agriculture and the use of modern agricultural methods including climate change. The gender of the household head does not determine the participation in urban agriculture. This does not in line with the finding by Kerr et al. [51] that gender is a determinant of taking up agricultural activity under climatic stressors as within the context of climate change adaptation strategies, ladies get climate information from informal networks more than men. Besides, urban agricultural production is influenced by household size. Literature has indicated the influence of household size and participation in agriculture [54,55]. Substantially, the understanding is that the larger the household size the greater the available labour, and the smaller the household size, the smaller the labour available to work, particularly on family farms [55]. In contrast, the relationship between people's participation in urban food production and their household income is statistically insignificant. This also supports the argument that socio-economic factors influence individual participation in agricultural production [56].

### 5.3 Socio-economic determinants of participation in urban food production

The participation in UA can be influenced by several socio-economic factors, including household income and size. This discussion explores the significant relationship between these factors and urban households' engagement in UA, particularly in the context of climatic stressors. In the age of climate change and variability, the size of an urban household is a determining factor for engaging in urban agriculture production, with an alpha level of 0.05 (p-value = 0.011 < 0.05) for households consisting of one to five persons. Larger households typically have more available labour, which can be a significant asset in UA. More family members can contribute to the various tasks involved in agriculture, from planting and watering to harvesting and selling produce. This labour availability can be particularly advantageous under climatic stressors, as it allows for more intensive management and care of crops. This does not support the argument that informal training of farmers has a positive association with agricultural productivity Chand [49], even though educated farmers are technologically advanced enough to take up climate change adaptation strategies [53,57]. Larger households have higher food demands, which can drive greater participation in UA as a means to supplement their food supply. This increased participation can be a direct response to climatic stressors that threaten food availability and affordability in urban areas. It is widely known that larger household size means the availability of labour to undertake agricultural productive activities relating to climate. This is in line with the work of Khanna and Sharma [58] that in agricultural production, labour is needed to prepare the land, to control weeds and for harvesting of the produce. It is for this reason that, Chandio et al. [59] all saw labour being significant in agricultural production activities among farmers. As such the results agreed with the assertion by Abdullah et al. [54] and Acevedo et al. [60] that household size is a determinant factor in the household member participation in agricultural activities. In larger households, there is often a broader base of agricultural knowledge and skills. Multiple family members may bring diverse experiences and knowledge about different farming techniques, which can be crucial for adapting to climatic stressors. The pooling of this knowledge can lead to more effective and innovative approaches to UA. This supports the work of Acevedo et al. [60] who said farmers' ages do not matter in their bid to scale up agricultural production adaptation to climate change but rather financial resources is the ultimate. Larger households can benefit from economies of scale in UA. They can manage larger plots of land more efficiently, share tools and resources, and collectively invest in necessary infrastructure. This collaborative approach can mitigate the impacts of climatic stressors by spreading the workload and sharing the risk among more individuals [26].

Furthermore, the results brought argument along the line of household income and their involvement in the production of urban agriculture in the context of climate change. The monthly income of an urban household determines the household's participation in urban agricultural production activity as households with household income < GHS1000.00 are more likely to engage in urban agriculture. Higher household income generally provides greater access to resources necessary for engaging in UA, such as land, seeds, tools, and water. Wealthier households can invest in better-quality inputs and technologies, which can enhance agricultural productivity even under climatic stressors like droughts or extreme temperatures. Therefore, the results corroborate the work of Rogito et al. [57] that people without the needed financial muscle and access to credit will not engage in agricultural production. Higher-income households may perceive UA as a supplementary activity rather than a primary source of livelihood. This perception allows them to absorb risks associated with climatic stressors more comfortably. They might also have the means to invest in adaptive measures such as irrigation systems, greenhouses, or other protective structures that lower-income households cannot afford. The study also supports the work of Lindblom et al. [61] who posited that farmers need funds to undertake sustainable agricultural intensification which is a feature of urban agricultural production under climate stressors. For lower-income households, UA can be a critical means of livelihood diversification and food security. Under climatic stressors, these households might increase their participation in UA as a coping strategy to ensure a stable food supply and reduce their dependency on market-bought food, which can become expensive or scarce during adverse weather conditions. Again, income levels can influence access to government or non-government support programs aimed at promoting UA. Often, wealthier households have better information and access to these programs, which can include financial incentives, training, and subsidies for climate-resilient agricultural practices.

The relationship between household income and size can have synergistic effects on UA participation. For instance, a larger, higher-income household can leverage both financial resources and labour availability to optimize its agricultural activities. They can invest in climate-resilient infrastructure and technologies while effectively managing labour-intensive tasks. Households with both higher income and larger size tend to have greater adaptive capacity. They can better withstand and adapt to climatic stressors due to their ability to invest in adaptive measures and mobilize labour effectively. This dual advantage makes them more resilient compared to smaller or lower-income households. It is important to note the variability among households. Some low-income but large households might still engage robustly in UA due to their need for food security and the availability of labour. Conversely, some high-income but small households might participate less due to the lack of labour, despite having financial resources.

## 6 Conclusions, recommendations, and policy implications

The study examined the socio-economic characteristics that determine an urban household's engagement in urban agricultural production. The study concluded that urban household size and monthly income are influential factors in urban households' participation in urban agricultural production. Understanding these determinants can provide valuable insights into why individuals in urban households' choose to engage in urban agriculture and can inform policies and strategies to support and promote sustainable urban agricultural practices. The study suggests that policymakers, urban planners, and community stakeholders should harness the potential of urban agriculture for sustainable development in the climate change era. This should be done through rolling out pro-poor urban development policies. These policies should include, but are not limited to, pro-poor rights and legislation in urban areas; poor access to financial markets; and land tenure reforms that include flexible land holding and access by the poor. However, more research should concentrate on the financing methods for urban farmers' adaptation plans to climate change as well as their degree of climate change awareness.

## 7 Limitations of the study

The study is limited in the following ways: the climate change adaptation knowledge of urban farmers was not measured. The study is also limited in that it does not look at factors other than the socio-economic factors of urban households.

## Ethics declaration

The manuscript is part of a Ph.D. thesis executed by the first author under the supervision of the second and third authors. There was no ethical review of the study. However, the data collection instruments for the manuscript were given full ethical consideration through expert review by two full professors of the Simon Diedong Dombo University of Business and Integrated Development Studies, Wa, Ghana.

Additionally, approval was granted from the head of the department for the environment and resource studies department at the Simon Diedong Dombo University of Business and Integrated Development Studies, Wa, Ghana, to send an introductory letter requesting cooperation from research participants with the survey results. The letter has been included in the proposal for further consideration. Every research subject gave their informed consent before any data collection. Additionally, it was guaranteed that study participants would be willing to stop participating.

## Funding

This research received no specific grant from funding agencies in the public, commercial, or not-for-profit sectors.

## Data availability

Data will be made available upon request.

## Additional information

No additional information is available for this paper.

## Informed consent statement

.

## Funding

.

## CRediT authorship contribution statement

**Godwin K. Naazie:** Visualization, Methodology, Formal analysis, Data curation, Conceptualization. **Isaac Agyemang:** Writing – review & editing, Supervision. **Anthony M. Tampah-Naah:** Writing – review & editing, Supervision.

## Declaration of competing interest

There is no competing interest among the Authors of this paper.
