# Supplementary material for: Our cities, our farm lands: The socioeconomic determinants of urban households participation in urban agricultural production under climatic stressors
Source: Heliyon. 2024 Aug 8;10(16):e35539. doi: 10.1016/j.heliyon.2024.e35539 (PMC11366882; doi:10.1016/j.heliyon.2024.e35539)
Supplement: Multimedia component 1 [file mmc1.docx]

**SIMON DIEDONG DOMBO UNIVERSITY OF BUSINESS AND INTEGRATED DEVELOPMENT STUDIES**

**FACUTY OF INTEGRATED DEVELOPMENT STUDIES**

**DEPARTMENT OF ENVIRONMENT AND RESOURCE MANAGEMENT**

**POST OFFICE BOX WA64, WA, UPPER WEST REGION – GHANA.**

**DOCTOR OF PHILOSOPHY (PHD) IN ENVIRONMENT AND RESOURCE MANAGEMENT**

**QUESTIONNAIRE FOR HOUSEHOLDS SURVEY**

***NB: Please answer by ticking or writing in the space provided***

| My name is ………………. I am a Doctor of Philosophy (PhD) student of the **Simon Diedong Dombo University of Business and Integrated Development Studies (SDD-UBIDS)**, Faculty of Integrated Development Studies, Department of Environment and Resource Studies. I am conducting my study on the topic, ***“Adaptation of Urban Agriculture to Climate Change and Variability Stressors in the Upper West Region, Ghana”.***. Participating in this exercise is voluntary and anything that would be said would be treated with high level of confidentiality. This questioning may last for about 45 minutes. Are you willing to respond, If yes, proceed. If no, approach another prospective respondents | | | | | |
| --- | --- | --- | --- | --- | --- |
| **Questions and Codes** | | **Options/code** | | | **Skip pattern/notes(if applicable)** |
| **SECTION A: CATEGORY AND LOCATION OF RESPONDENTS INTERVIEWER DETAILS** | | | | | |
| **A1:Interviewer’s name** | | ……………………………………………………… | | |  |
| **A2:Interviewer ID** | | …………………………………………………….. | | |  |
| **A3:** Class/zone of residents | | 1. Zone 1 [ ] 2. Zone 2 [ ] 3. Zone 3 [ ] | | |  |
| **A4:** Household name | | …………………………. | | |  |
| **A5:** Household number | | …………………….. | | |  |
| **SECTION B: SOCIO-ECONOMIC AND DEMOGRAPHIC CHARACTERISTICS** | | | | | |
| ***NB: In this section, I would like to ask your questions about your social and economic life as well as households information*** | | | | | |
| **B1:** Age of the respondents | | | ……………………………. | |  |
| **B2:** Gender of the respondents | | | 0. Male [ ]  1. Female [ ] | |  |
| **B3:** Marital status | | | 1. Single [ ] 2. Married [ ] 3. Separated [ ] 4. Widowed [ ] 5. Others, specify ……………. | |  |
| **B4:** Employment status | | | 1. Employed [ ] 2. Unemployed [ ] | |  |
| **B5:** Type of employment | | | 1. Formal worker [ ] 2. Informal worker [ ] | |  |
| **B6:** Education attainment | | | 1. No Formal Education [ ] 2. Basic Education [ ] 3. SHS/Secondary Education [ ] 4. Tertiary Education [ ] | |  |
| **B7:** Residential status (settlers or natives) | | | 1. Native [ ] 2. Settler [ ] | |  |
| **B8:** Households incomes level | | | ……………….. | |  |
| **B9:** Household size | | | ……………………… | |  |
| **SECTION C: CHARACTERISTICS OF URBAN FARMING** | | | | | |
| ***NB: In this section, I would like to ask you few question about the features of your agriculture production in the urban space (crop or animals/both)*** | | | | | |
| **C1: Do you engage in UA** | | | 1. Yes [ ] 2. No [ ] | | ***If yes, skip C2 to C4, if no complete C2 to C4 and F1 to F26*** |
| **C2:** If no to question **C1,** which of the following affect your participation in UA ***(tick as many as possible)*** | | | 1. Access to land [ ] 2. Inability to control animals’ destructions [ ] 3. Low yield due to weather changes [ ] 4. Less availability of knowledge [ ] 5. Land tenure security [ ] 6. Difficulties in the use of public lands (legal actions and/or confiscation) [ ] 7. Access and affordability of inputs [ ] 8. No interest [ ] 9. Others, specify……………………………………………. | |  |
| **C3:** Despite your no participation in UA, how will you rate the relevance of participation in UA | | | 1. Very relevant [ ] 2. Relevant [ ] 3. No idea [ ] 4. Very irrelevant [ ] 5. Ireelevant [ ] | |  |
| **C4:** Which of the following explains your ratings in question C3 above .***(tick as many as possible)*** | | | 1. Addition to households’ income [ ] 2. Supplement to urban households’ food needs[ ] 3. Employment to people who practice it [ ] 4. Environmental benefits (e.g, beautification, carbon sink, solid wastes and wastes water treatment, etc.) [ ] 5. Reduction in distances to get food [ ] 6. Promotes all year consumption of fresh vegetables [ ] 7. Others, specify …………………………………….. | |  |
| **C5:** If you participate in UA, which of the following described your land tenure arrangement ? | | | 1. Family/relative land [ ] 2. Purchased [ ] 3. Gift [ ] 4. Rented [ ] 5. Other………………………………… | |  |
| **C6:** How will you describe land accessibility with respect to your participation in UA | | | 1. Easy to access land [ ] 2. Difficult to access land [ ] | |  |
| **C7:** What is the main purpose of acquiring the land that is now for UA? | | | 1. For residential purpose [ ] 2. For farming purpose [ ] 3. For educational purpose [ ] 4. For commercial purpose [ ] 5. For religious purpose [ ] 6. Others, specify …………………… | |  |
| **C8:** Type of urban agriculture | | | 1. Crop production [ ] 2. Animals production [ ] 3. Both crop and animal production [ ] | | ***If option 0, skip QC9 – C25a;***  ***If option 1, skip QC3 – C9; If option 2, no skip*** |
| **C9:** Location of Urban farm | | | 1. Roadside [ ] 2. Riverside [ ] 3. Park [ ] 4. Public land [ ] 5. Private residential [ ] 6. Industrial [ ] 7. Homes [ ] 8. Uncompleted building [ ] 9. Other, specify …………………………………… | |  |
| **C10:** How long have you been into crop cultivation in the urban area in years? | | | ……………………….. | |  |
| **C11:** What is the size of your plot for crop cultivation? (in acres) | | | …………………………….. | |  |
| **C12:** Is the farm fenced? | | | 1. Yes [ ] 2. No [ ] | |  |
| **C12a:** If yes, what materials are used in fencing? | | | 1. Block/bricks [ ] 2. Wire mesh [ ] 3. Sticks [ ] 4. Guinea corn stocks [ ] 5. Others, specify | |  |
| **C13:** What type of crop do you grow? (***tick as many as possible***) | | | 1. Maize [ ] 2. Millets [ ] 3. Beans [ ] 4. Groundnuts [ ] 5. Yams [ ] 6. Cassava [ ] 7. Okara [ ] 8. Tomatoes [ ] 9. Pepper [ ] 10. Cabbage [ ] 11. Pumpkin leaves [ ] 12. Other specify ……………………………….. | |  |
| **C14:** What motivates your interest in crop cultivation? | | | 1. Sale for income [ ] 2. To meet household food needs [ ] 3. Just to occupy the land for protection [ ] 4. Leisure [ ] 5. Other, specify ………………………………. | |  |
| **C15:** How often do you involve in the activities in the urban farm? | | | 1. Everyday [ ] 2. Once a week [ ] 3. Once a month[ ] 4. Twice a week [ ] 5. Twice a month [ ] | |  |
| **C16:** What is the main source of labour to your crop farming? | | | 1. Hired labour [ ] 2. Family labour [ ] 3. Social labour [ ] 4. Other specify ……………………………………. | |  |
| **C16a:** If hired labour, how much do you spend per season? | | | ………………………….. | |  |
| **C17:** How much do you spend on inputs? | | | ……………………… | |  |
| **C18:** What is the main purpose for cultivating crops in the urban area? | | | 1. For sale only [ ] 2. For consumption only [ ] 3. For consumption with minimal sales [ ] | |  |
| **C18a:** If option 1 or 3 in Q 17, how much do you get per season? | | | ……………….. | |  |
| **C19:** What type of animals do you rear? (***tick as many as possible***) | | | 1. Chicken [ ] 2. Guinea fowls [ ] 3. Ducks [ ] 4. Turkeys [ ] 5. Goats [ ] 6. Sheep [ ] 7. Cattle [ ] 8. Pigs [ ] 9. Others, specify ………………………………. | |  |
| **C20:** What is the size of your plot for rearing? | | | ………………….. | |  |
| **C21:** How do you keep your animals? | | | 1. Intensive [ ] 2. Semi-intensive [ ] 3. Free range [ ] | |  |
| **C22:** Do you house your animals separately? | | | 1. Yes [ ] 2. No [ ] | |  |
| **C22a:** If yes, what unit do you house poultry? | | | 1. Bricks/Blocks Built unit [ ] 2. Wooden structure [ ] 3. Open space [ ] 4. Others, specify | |  |
| **C22b:** If yes, what unit do you house ruminants? | | | 1. Bricks/Blocks Built unit [ ] 2. Wooden structure [ ] 3. Open space [ ] | |  |
| **C22c:** If yes to **C22**, what unit do you housE pigs? | | | 1. Bricks/Blocks Built unit [ ] 2. Wooden structure [ ] 3. Open space [ ] | |  |
| **C23:** What is the reason for your choices in either **C22a, C22b and/or C22c C20c** above? | | | 1. To prevent theft [ ] 2. Easy to reach animals [ ] 3. To prevent disease infections [ ] 4. Others specify ………………………………… | |  |
| **C24:** What motivates you into rearing? | | | 1. Sale for income [ ] 2. To meet household nutritional needs [ ] 3. Just to occupy the land for protection [ ] 4. Leisure [ ] 5. Other specify ………………………………………… | |  |
| **C25:** How often do you involve in rearing activities in the urban area? | | | 1. Everyday [ ] 2. Once a week [ ] 3. Once a month [ ] 4. Twice a week [ ] 5. Twice a month [ ] 6. Others, specify | |  |
| **C26:** What is the main source of labour to your animals rearing? | | | 1. Hired labour [ ] 2. Family labour [ ] 3. Social labour [ ] 4. Others, specify | |  |
| **C26a:** If hired labour, how much do you spend per season? | | | ………………….…………. | |  |
| **C27:** How much do you spend on inputs? | | | ………………………. | |  |
| **C28:** how do you consider rearing animals in the urban area? | | | 1. As part-time [ ] 2. As full time [ ] | |  |
| **C28a:** If option 1 or 3 in ***QC25*** how much do you get per season? | | | ………………………….. | |  |
| **SECTION D: THE EFFECTS OF LAND USE CHANGE ON RESIDENTS’ PARTICIPATION IN URBAN AGRICULTURE (*SATELLITE IMAGES*)** | | | | | |
|  |  | | | |  |
|  |  | | | |  |
|  |  | | | |  |
| **SECTION E: TO EXPLORE CLIMATE CHANGE ADAPTATION STRATEGIES OF URBAN FARMERS** | | | | | |
| **NB: In this section, I would like to ask you questions on how you adapt your crop farming and animals rearing to the changing weather** | | | | | |
| **E1:** Do you have any knowledge in climate change? | | | 1. Yes [ ] 2. No [ ] | |  |
| **E1a:** If yes in ***E1***, what is climate change about? | | | ………………………………….. | |  |
| **E1b:**If yes to ***E1,*** how do you get the climate information? | | | 1. From friends and neighbors [ ] 2. From agriculture services workers (MoFA/NGOs) [ ] 3. Television [ ] 4. Radio [ ] 5. Personal observation [ ] 6. None [ ] 7. Others specify …………………. | |  |
| **E2:** Isclimate change affecting your crops cultivation or animal rearing? | | | 1. Yes [ ] 2. No [ ] | |  |
| **Eaa:** If yes to ***E3*** above*,* in what ways does climate affect your activities? ***(choose as many as possible)*** | | | 1. Insufficient water [ ] 2. Low yields [ ] 3. Pest and diseases infestation [ ] 4. Erosion effects [ ] 5. Nutrients leaching [ ] 6. Post- harvest losses [ ] 7. Others specify ……………………………………. | |  |
| **Ebb:** If yes to ***E3*** above***,*** how do you manage weather changes in crop production? ***(Choose as many as possible)*** | | | 1. Growing early maturing crops [ ] 2. Shifting planting dates [ ] 3. Mixed cropping [ ] 4. Irrigation [ ] 5. Others, specify | |  |
| **E3:** What is the main source of water for your crop cultivation? ***(Choose as many as possible)*** | | | 1. Rain [ ] 2. Drains, streams, small rivers, dug-out/wells [ ] 3. Pipe-borne water from public water system [ ] 4. Mechanized boreholes [ ] 5. Others specify ………………………………… | |  |
| **E4:** What is the main land preparation practices in your crop cultivation? | | | 1. Raised mounds [ ] 2. Zero tillage [ ] 3. Tractor ploughing [ ] 4. The use of ridges [ ] 5. The use of hand [ ] 6. Others specify ……………………………………… | |  |
| **E5:** What are the farm implements used in your crop farming ***(choose as many as possible)*** | | | 1. Hoes [ ] 2. Tractor [ ] 3. Planter [ ] 4. Thrasher [ ] 5. Cutlasses [ ] 6. Combine harvesters [ ] 7. Other specify ……………………………………………… | |  |
| **E6:** What soil and water management practices do you use? ***(choose as many as possible)*** | | | 1. Mulching [ ] 2. Intercropping [ ] 3. Cover cropping [ ] 4. Ridging [ ] 5. Tired ridging [ ] 6. Other specify ……………………………………………… | |  |
| **E7:** What soil erosion management practices do you apply to your farm? ***(choose as many as possible)*** | | | 1. Boundary bounds [ ] 2. Contour ploughing [ ] 3. Stone bonding [ ] 4. Cover cropping [ ] 5. Other specify ……………………………………………… | |  |
| **E8:** What soil fertility management practices do you practice? (***tick as many as possible***) | | | 1. Application of mineral fertilizers [ ] 2. The use of crop residues [ ] 3. Manure [ ] 4. Compost [ ] 5. Other specify ……………………………………………… | |  |
| **E9:** How do you manage weather changes in crop production? | | | 1. Growing early maturing crops [ ] 2. Shifting planting dates [ ] 3. Mixed cropping [ ] 4. Irrigation [ ] 5. Others, specify …………….. | |  |
| **E10:** What type/variety of seeds do you sowin your farm? | | | 1. Local/indigenous seeds [ ] 2. Improved seeds [ ] 3. Hybrid seeds [ ] 4. Both improved and indigenous/local seeds [ ] 5. Others, specify ………………………………………. | |  |
| **E11:** What is the sources of seeds you grow? | | | 1. Own seeds [ ] 2. Bought from agro-shops [ ] 3. Family/relative seeds [ ] | |  |
| **E12:** What is the yield situation of your crops? | | | 1. Good [ ] 2. Average [ ] 3. Not good [ ] | |  |
| **E13:** Do you ever benefit from the services of Agricultural Extension Services from MoFA or any organization ? | | | 1. Yes [ ] 2. No [ ] | |  |
| **E13a:** If yes to ***E14*** above, how often? | | | 1. Very often [ ] 2. Often [ ] 3. Sometimes [ ] 4. Not all [ ] | |  |
| **E14:** What can you say about the production level of your crops amid weather changes? | | | 1. Good [ ] 2. Average [ ] 3. Not good [ ] | |  |
| ***ANIMALS*** | | |  | |  |
| **E15:** What breed of animals do you rear? | | | 1. Indigenous/local [ ] 2. Exotic/foreign [ ] 3. Both Indigenous/local and Exotic/foreign [ ] | |  |
| **E16:** What is the main sources of feeds to your animals? | | | 1. Own feed [ ] 2. Purchase seeds from the market [ ] 3. Gift from friend/ relatives [ ] | |  |
| **E16a:** If option 1 in ***E16*** above***,*** from which source do you buy the feed from? | | | 1. Certified feed dealers [ ] 2. Open market [ ] 3. Other sources, specify, ……………………………….. | |  |
| **E17:** How do you house your animals against bad weather? | | | 1. In rooms with light and air [ ] 2. In rooms without light but aerated [ ] 3. In wooden structure with light and aerated [ ] 4. In a wooden structure without light but aerated [ ] | |  |
| **E18:** Do you treat your animals? | | | 1. Yes [ ] 2. No [ ] | |  |
| **E18a:** If yes to ***E19*** above, how often? | | | 1. Very often [ ] 2. Often [ ] 3. Sometimes [ ] 4. Not all [ ] | |  |
| **E18b:** If yes to ***E19,*** how often? | | | 1. Very often [ ] 2. Often [ ] 3. Sometimes [ ] 4. Not all [ ] | |  |
| **E18c:** If yes to ***E19,*** which source do you get the treatment from? | | | 1. Veterinary Department of MoFA [ ] 2. Self-medication [ ] 3. Private Veterinary practitioner [ ] 4. Other, specify, ……………… | |  |
| **E18d:** IF yes to ***E19***, in which of the following ways? | | | 1. Vaccination 2. Giving the animals pills 3. Disinfecting pens 4. Spraying animals with some insecticides 5. Other, specify, ……………………………………… | |  |
| **E18e:** if yes to ***E19,*** how? | | | ­­­­­­­­­­­­­…………………………………………………. | |  |
| **E18e:** If no to ***E19,*** how do you protect your animals from diseases? | | | ………………………………………………… | |  |
| **E19:** What is the main source of information on weather changes to guide your animals rearing? | | | 1. From friends and neighbors [ ] 2. From agriculture services workers (MoFA/NGOs) [ ] 3. Television [ ] 4. Radio [ ] 5. None [ ] 6. Personal observation [ ] | |  |
| **E20:** What do you have to say about your animals production amid weather changes? | | | 1. Good [ ] 2. Average [ ] 3. Not good [ ] | |  |
| **SECTION F: URBAN AGRICULTURE CONTRIBUTIONS TO HOUSEHOLDS’ FOOD SECURITY** | | | | | |
| ***NB: Food security: At this point, I would like to ask you questions relating to food security in your households.*** | | | | | |
| **F1:** Do you think community garden changed your lifestyle? | | | | 1. Yes [ ] 2. No [ ] | ***NB: For both urban and non-urban farmers*** |
| **F2:** If yes, why? | | | | 1. Yes, I feel eating healthier [ ] 2. Yes, I save more money [ ] 3. Yes, I feel it is more convenient [ ] 4. Yes, other reasons   ……………………………………… |  |
| **F3:** If no, why? | | | | 1. No, I always live like this [ ] 2. No, I don’t feel much differences [ ] 3. No, other reasons   …………………………………………… |  |
| ***Food availability*** | | | |  |  |
| **F4:** Is urban farming the main source of food supply to your household? | | | | 1. Yes [ ] 2. No [ ] |  |
| **F4a:** If yes to ***F4*** did you or other household members ever skip a meal because there was no enough money to buy food over the last 12 months? | | | | 1. Once a week [ ] 2. Once a month [ ] 3. Once in 3 months [ ] 4. Once in 6 months [ ] 5. Never skipped the meal [ ] 6. Don’t know [ ] |  |
| **F5:** Is the produce from your urban farming enough for your household throughout the year? | | | | 1. Yes [ ] 2. No [ ] 3. Sometimes [ ] |  |
| **F6:** Has your household been able to feed on produce from urban farming in the past 12 months? | | | | 1. Yes [ ] 2. No [ ] 3. Don’t know [ ] |  |
| **F7:** Did you ever cut the size of children’s meals due to no enough food available? | | | | 1. Once a week [ ] 2. Once a month [ ] 3. Once in 3 months [ ] 4. Once in 6 months [ ] 5. Never [ ] 6. Don’t know [ ] |  |
| ***Food accessibility*** | | | |  |  |
| **F8:** Does your household get food from your urban farming throughout the years? | | | | 1. Yes [ ] 2. No [ ] 3. Sometimes [ ] |  |
| **F9:** How often did you or other household members ever not eat for a whole day because of lack of food in the house? | | | | 1. Once a week [ ] 2. Once a month [ ] 3. Once in 3 months [ ] 4. Once in 6 months [ ] 5. Never happened [ ] 6. Don’t know [ ] |  |
| **F10:** Did any of the children ever skip a meal due to no enough food in the house? | | | | 1. Once a week [ ] 2. Once a month [ ] 3. Once in 3 months [ ] 4. Once in 6 months [ ] 5. Never [ ] 6. Don’t know [ ] |  |
| **F11:** Did any of the children ever not eat for a whole day because of lack of money to  buy food? | | | | 1. Once a week [ ] 2. Once a month [ ] 3. Once in 3 months [ ] 4. Once in 6 months [ ] 5. Never [ ] 6. Don’t know [ ] |  |
| ***Food affordability*** | | | |  |  |
| **F12:** Apart from your urban farm produced, is your household able to afford other food produced in the urban center? | | | | 1. Yes [ ] 2. No [ ] 3. Sometimes [ ] |  |
| **F13:** Are vegetables produced in the urban space expensive? | | | | 1. Yes [ ] 2. No [ ] 3. Sometimes [ ] |  |
| **F14:** Are animals products like ruminants produced in the urban centers | | | | 1. Yes [ ] 2. No [ ] 3. Sometimes [ ] |  |
| **F15:** Are you able to buy chicken produced in the urban centers? | | | | 1. Yes [ ] 2. No [ ] 3. Sometimes [ ] |  |
| **F16:** In your own view which of these produced in the urban space are costly? | | | | 1. Chicken and chicken products [ ] 2. Small ruminants [ ] 3. Vegetables [ ] |  |
| ***Food utilization*** | | | |  |  |
| **F17:** Do you think your crop farming in the urban center has improved your household’s daily food intake? | | | | 1. Yes [ ] 2. No [ ] |  |
| **F17a:** If yes, to what extent? | | | | 1. Slightly better [ ] 2. Better [ ] 3. Much better [ ] |  |
| **F17b:** If no, then | | | | 1. Same [ ] 2. Worse [ ] 3. Much worse [ ] 4. Don’t know |  |
| **F18:** How long has your household been able to feed on your own urban food produce? | | | | 1. Less than 3 months [ ] 2. 3 months [ ] 3. More than 3 months [ ] 4. 6 months [ ] 5. The whole year [ ] |  |
| **F19:** How many times do your household eat in a day? | | | | 1. Once [ ] 2. Twice [ ] 3. Thrice [ ] 4. None [ ] |  |
| **F20:** In the past one month has any member of your household skip meal | | | | 1. Yes [ ] 2. No [ ] |  |
| **F20a:** If yes, what was the reason? | | | | 1. Unavailability of food [ ] 2. Voluntarily [ ] 3. As a way of managing food [ ] |  |
| **F21:** In the past one week, has your household face with food challenges? | | | | 1. Yes [ ] 2. No [ ] |  |
| **F22:** In the past, 3 months, has your household faced food challenges? | | | | 1. Yes [ ] 2. No [ ] |  |
| **F23:** In the past 6 months, has your household faced food challenges? | | | | 1. Yes [ ] 2. No [ ] |  |
| **F24:** In the past one year, has your household faced with food challenges? | | | | 1. Yes [ ] 2. No [ ] |  |
| ***Coping strategies*** | | | |  |  |
| **F25:** Did you or other household members ever eat less than you or they would have needed to eat? | | | | 1. Once a week [ ] 2. Once a month [ ] 3. Once in 3 months [ ] 4. Once in 6 months [ ] 5. Never [ ] 6. Don’t know [ ] |  |
| **F26:** In case of food shortage in your household, how do you dealt with it? ***(tick all apply)*** | | | | 1. Borrowing from friends, neighbors, relatives, etc. [ ] 2. Reduce frequency of eating in a day by all household members [ ] 3. Reduce expenditures on health [ ] 4. Reduce expenditures on education [ ] 5. Adults skip meals once a day [ ] 6. Selling assets (e.g. bike cycle, motorcycle, bull, farm equipment, etc) [ ] 7. Others (Please, specify………………………… |  |

***END OF THE QUESTIONNAIRE!!!!!!!!!!***
